# The frequency, clinical course, and health related quality of life in adults with Gilbert’s syndrome: a longitudinal study

**DOI:** 10.1186/s12876-019-0931-2

**Published:** 2019-02-04

**Authors:** Sanaa Kamal, Sara Abdelhakam, Dalia Ghoraba, Yasmin Massoud, Kareem Abdel Aziz, Huda Hassan, Tamer Hafez, Ahmed Abdel Sallam

**Affiliations:** 1 Department of Tropical Medicine and Gastroenterology, Ain Shams Faculty of Medicine, Ain Shams University, Abbassia, Cairo, 11341 Egypt; 2 Department of Clinical Pathology, Cairo University, Cairo, Egypt; 3 Department of Molecular Biology and Genetics, The American University, Cairo, Egypt; 4 Faculty of Medicine, Misr University for Science and Technology, Cairo, Egypt

**Keywords:** Hyperbilirubinemia, Gilbert syndrome, *UGT1A1* polymorphisms, Health related quality of life

## Abstract

**Background:**

Gilbert syndrome (GS) is an autosomal recessive inherited disorder of bilirubin glucuronidation which has not been investigated in Egypt. This longitudinal study investigated the frequency, clinical course, genetic profile and health related quality of life in Egyptian adults.

**Methods:**

An initial cross-sectional study was conducted to assess the frequency of Gilbert syndrome among Egyptian adults. Subjects fulfilling the criteria of GS were enrolled into the study and prospectively followed for the clinical features, risk factors for hyperbilirubinemia, health related quality of life [Short form-36 Health Survey version 2 (SF-36v2) and Chronic Liver Disease Questionnaire (CLDQ)], vitamins assessment and UGT1A1 polymorphisms.

**Results:**

One hundred and one subjects fulfilled the criteria of GS with a prevalence of 8.016%. Recurrent jaundice was the only presentation in 47 (56.627%) GS subjects and jaundice was associated with abdominal pain, dyspepsia or loss of appetite in 54 (53.465%) subjects. A significant difference in 25-Hydroxyvitamin D3 levels was detected between GS patients and control subjects (*P* <  00001). Twelve subjects with GS developed significant unconjugated bilirubinemia during direct antiviral therapy (DAAs) therapy for HCV despite achieving sustained virologic response. Pregnancy was associated with significant exacerbation of unconjugated bilirubin which persisted through pregnancy. Risk factors for clinical jaundice included general anesthesia, pregnancy, fasting > 12 h, pregnancy, and low calorie weight losing plans, systemic infections, and intensive physical effort. During jaundice attacks, subjects with GS had significant differences in vitality, role emotional, social functioning, worry and general health domains of the SF-36v2 and CLDQ compared to controls. The homozygous polymorphism A(TA)7TAA was the most frequent polymorphism in Egyptians with GS.

**Conclusion:**

Gilbert syndrome is a frequent inherited disorder in Egypt. In a substantial percentage of subjects with GS, episodes of jaundice are associated with other symptoms and nutritional deficiencies which result in impairment of HRQOL. Screening, counseling, monitoring and individualized health care are recommended for subjects with GS in the setting of anesthesia, pregnancy, treatment with DAAs, deliveries, surgery and weight loss plans.

## Background

Gilbert’s syndrome (GS) is an autosomal recessive inherited disorder of bilirubin glucuronidation characterized by unconjugated hyperbilirubinaemia in the absence of hepatocellular injury or hemolysis [1–4]. Gilbert syndrome is characterized by mild, chronic, unconjugated hyperbilirubinemia in the absence of hepatic injury or overt hemolysis [5, 6]. GS has a worldwide distribution, but prevalence rates vary among different populations and ethnic backgrounds. Overall, some studies estimated that GS is found in approximately 3–10% of the general population [6, 7]. GS is present at birth, but may remain undiagnosed until the second decade of life where subjects develop mild hyperbilirubinaemia or may present with fluctuating overt jaundice [5–7], Gilbert syndrome has been shown to be associated with symptoms in patients when treated with some medicines such as irinotecan [7].

Gilbert’s syndrome is characterized by mutations to the UGT1A1 gene located on the long arm (q) of chromosome 2 (2q37) which encodes the enzyme uridine disphosphate-glucuronosyltransferase-1A1 (UGT1A1) which is required for the conjugation and subsequent excretion of bilirubin [8–10]. Several *UGT1A1* variants have been reported including UGT1A1*28, UGT1A1*60 and UGT1A1*93. UGT1A1*28, the frequent variant among Caucasians. TA repeat expansion in the promoter of *UGT1A1* with an increase in the number of TA dinucleotides from 6 to 7 repeats [8, 9]. The homozygous polymorphism [A(TA)7TAA] in the promoter of the gene for UDP-glucuronosyltransferase 1A1 (UGT1A1) is the most frequent genotype of Gilbert’s syndrome [11].

Gilbert syndrome is an ignored clinical entity in many countries as only few studies investigated the frequency and the clinico-genetic patterns of this disorder [1, 3]. In Egypt, no studies have been conducted to investigate any epidemiologic, clinical or genetic aspects of GS. This may be due to the high prevalence of other diseases presenting with various forms of hyperbilirubinemia such as viral hepatitis, cholelithiasis and thalassemia. Thus, we conducted the current study to investigate the frequency, clinical features, nutritional status, risk factors for hyperbilirubinemia and genetic patterns and the health*-*related quality of life (HR-QOL) in Egyptian patients with Gilbert syndrome. Given the high prevalence of hepatitis C in Egypt, we also investigated the clinical presentations of patients with concomitant Gilbert syndrome and HCV after successfully eradicating HCV infection.

## Methods

### Study design and study cohorts

The current study consists of an initial cross-sectional screening phase followed by a prospective, longitudinal phase. The study was conducted in several Egyptian centers in Cairo, Delta and Upper Egypt (Ain Shams University Hospitals, Cairo, Misr University for Science and Technology Faculty of Medicine, and Gastroenterology centers in Cairo and Minya) from September, 2011 through December, 2017. The study protocol and patients’ informed consent were approved by the review boards of the participating board prior to the commencement of this study. All patients signed a written informed consent before enrollment in the study and before any procedure related to the study. The patients’ anonymity be carefully protected through the entire study. The study was conducted in accordance with the Declaration of Helsinki and was consistent with the International Conference on Harmonization and Good Clinical Practice.

The initial cross-sectional survey consisted of an online survey that was administered to a sample representative of the Egyptian population aged between 16 and 45 years applying quota sampling approach. Questionnaires were sent either by email, social media (whatsapp, face book etc) or distributed by hand and filed at universities, schools, workplaces, clinics, hospitals. The first part of the survey consisted of open ended and closed ended questions concerned with the socio-demographic data such as age, sex, occupation and work experience. The second part of the questionnaire included questions about previous or current intermittent jaundice, any diseases or drug intake that maybe associated with jaundice, any laboratory evidence of hyperbilirubinemia, history or GS in the participant or his/her family.

Patients providing a history of diagnosed GS or intermittent jaundice episodes or history of investigations showing hyperbilirubinemia with elevated serum bilirubin were invited to join the study for further assessment. Patients were enrolled in the study if they fulfilled all the following criteria: *i)* history of recurrent episodes of intermittent jaundice; *ii)* detection of predominant unconjugated hyperbilirubinemia (more than 1 mg/dL) after fasting on several occasions; *iii)* absence of hemolytic disease, iv) absence of altered liver function tests (LFT) results (other than bilirubin); *iv)* absence of other disease processes that may cause jaundice, v) positive fasting test characterized by 2- to 3-fold rise in plasma unconjugated bilirubin level on fasting or consuming a diet of ≤400 kcalst, *vi)* positive rifampin test.

A group of patients with unconjugated hyperbilirubinemia and previous history of successfully treated hepatitis C infection or spontaneously resolved hepatitis C were identified and enrolled in the study if they fulfilled the criteria for GS in addition to undetectable HCV RNA titers confirmed by two real-time PCR assays for HCV (Cobas AmpliPrep/Cobas TaqMan instruments (Roche Diagnostics, Basel, Switzerland). One hundred age and gender matched healthy subjects were also enrolled as a control group. Enrolled patients were invited to complete an Arabic version of the chronic liver disease questionnaire (CLDQ) [12] and the Arabic Egyptian version of short form-36 Health Survey version 2 (SF-36v2) at entry, during the icteric episodes and the anicteric intervals [13].

### Provocative tests

#### Fasting test

A blood sample was taken from the individual for measuring total and unconjugated plasma bilirubin. After 24 h calorie restriction (300 kcal per day), the enrolled subject comes fasting and other blood samples are taken for total and unconjugated plasma bilirubin estimation. In normal individuals, total bilirubin concentrations rise by approximately 60% after 48 h fasting. In subjects with Gilbert’s syndrome, the total bilirubin concentration rises by approximately 90% after 24 h. The unconjugated bilirubin concentration rises by more than 110% after 24 h [14, 15].

#### Rifampin provocative test

The rifampin provocative test was performed to patients with suspected Gilbert’s syndrome as previously described [16]. Briefly, individuals were given 600 mg of rifampicin. Unconjugated bilirubin levels were measured before and four hours after rifampicin. A significant increase in mean unconjugated bilirubin levels suggests GS hyperbilirubinemia [16, 17].

##### Assessment of nutritional status and vitamins assays

The height, weight, and body mass index were measured at entry and follow up including icteric and anicteric periods. Vitamin B12 was measured using the Diazyme Vitamin B12 kit (Diazyme Laboratories, Inc., Poway, CA, USA) and vitamin D was measured using a 5-OH Vitamin D Test Kit (Diazyme Laboratories, Inc. CA, USA) according to the manufacturer’s instructions. Plasma folate levels were determined with a commercial automatic electrochemical immuno-analyzer (Roche E170) and electrochemiluminescence immunoassay (ECLIA) kit [18, 19].

The potential impact of certain foods on the bilirubin levels was investigated in individuals with and without GS. The study subject was given a prescribed two-week feeding period with a diet either supplemented or devoid of the specific food.. Bilirubin concentrations were measured at baseline and on days 7 and 15 after the start of the test diet.

##### UGT1A1 genotyping

Enrolled subjects were tested for uridine diphosphate*-*glucuronyl transferase 1A1 (UGT1A1)*28 genotype and promotor polymorphisms using the UGT1A1*28 Genotyping Kit (EntroGen, Inc., Woodland Hills, CA, USA) according to the manufacturer’s instructions. Briefly, the UGT1A1 Genotyping Kit is a polymerase chain reaction (PCR)-based assay that utilizes allele-specific probes to identify the common UGT1A1 polymorphic variants. The testing procedure involves DNA extraction, amplification of regions of the UGT1A1 gene using allele-specific probes and analysis using real-time PCR instrument software. The analysis included polymorphisms of the *UGT1A1* gene, including single nucleotide polymorphisms (SNPs) UGT1A1*6 (211G > A), UGT1A1*27 (686C > A), UGT1A1*60 (− 3263 T > A), and TA repeat variation of UGT1A1*28 (A(TA)_7_TAA) [20–22].

##### Health-related quality of life assessment

Patients were invited to complete the Arabic version of the Short form-36 Health Survey version 2 (SF-36v2) [12, 13] and Chronic Liver Disease (CLDQ) [23, 24] health survey questionnaires at several times points during the study (at entry, during jaundice attacks and between jaundice attacks). The data obtained from the SF36v2 questionnaires were used to classify patients into the SF-6D system which includes six attributes namely physical functioning (PF), role limitation (RL), social functioning (SF), bodily pain (PL), mental health (MH) and vitality (VT) in addition to a scoring table to compute the utilities [25–27].

The CLDQ consists of 29 items measuring six domains of abdominal symptoms (AS), fatigue (FA), systemic symptoms (SS), activity (AC), emotional function (EF) and worry (WO). Each item is rated on a 7-point Likert scale, with 1 indicating ‘all of the time’ and 7 indicating ‘none of the time’. An overall score is evaluated by calculating the mean of all domain scores. Each domain and the overall score range from 1 to 7, with a higher score indicating better HRQOL [23–27].

### Statistical analysis

Continuous variables were expressed as mean ± SD or median (range) as appropriate. Categorical variables were compared by Chi-square test or Fisher’s exact test as appropriate. Continuous variables were compared by *Student’s* t-test ANOVA or Mann–Whitney test as appropriate. Prevalence estimates and 95% confidence intervals (CI) were obtained with the statistical program CSAMPLE provided with EpiInfo 6.04b (Centers for Disease Control & Prevention, Atlanta, GA, USA), which allows correction of the variance for the design effect by adding variables for the stratum and for the primary sampling unit. Correlation between variables is performed by Pearson correlation for parametric and Spearman for non-parametric testing. Multiple regression analysis was performed by D’Agostino-Pearson test. Statistical analyses were performed by SPSS for Windows, version 22 (MedCalc Software, Ostend, Belgium); a *t*-test (http://www.vassarstats.net/tu.html), the Mann-Whitney *U*-test by using an online calculator (http://www.socscistatistics.com/tests/mannwhitney/Default2.aspx).

## Results

During the cross sectional screening phase of the study, 1260 Egyptians between the age of 14 and 45 years completed the online survey. Based on the results of the questionnaire and the clinical and laboratory criteria, 101 (8.016%) individuals fulfilled the criteria of GS. Extrapolating to the total Egyptian population (around 90 million), there are approximately 7 million GS cases in Egypt. Enrolled individuals were categorized into two groups: Group A which comprised 83 patients with GS and not history of HCV and Group B which comprised 18 patients with GS and successfully eradicated HCV (undetectable HCV-RNA). The demographics of the enrolled subjects are shown in (Table 1).

**Table 1:** Demographics of enrolled GS subjects and controls

Parameters	*Group A* Patients with Gilbert syndrome (*n* = 83)	*Group B* Patients with GS and eradicated HCV (*n* = 18)	Controls (*n* = 100)	*P* value
Age (years) mean ± SD (range)	28.1 ± 8.6 (18–39)	34.3 ± 9.5 (21–42)	31 ± 10.1 (21–42)	Group A vs. B: 0.0058 Group A vs Control: 0.1532 Group B vs control: 0.0285*
Male/Female	57/26	15/3	58/42	Group A vs. B: 0.2621 Group A vs Control: 0.1670 Group B vs. control: 0.0631
Parental consanguinity: n (%)	45 (54.217)	9 (50)	21 (21)	Group A vs. B: 0.7964 Group A vs Control: < 0.0001** Group B vs. control: < 0.0001**
Cigarette smoking; n (%)	6 (7.228)	4 (22.222)	26 (26)	Group A vs. B: 0.0750 Group A vs Control: 0.0008 Group B vs. control: 1.0000
BMI (kg/cm^2^) mean ± SD (range)	23.4184 ± 2.36 (18.6–26.7)	23.095 ± 2.9 (23–27)	25.5 ± 3.94 (19.8–27.4)	Group A vs. B: 0.6149 Group A vs control: < 0.0001** Group B vs. control: 0.0150*

### Characteristics of the enrolled GS cases

The clinical characteristics of the subjects with GS identified are shown in Table 2. The number of males with GS was higher than females. In the current study, GS was recognized more frequently in cities than in villages, with an estimated prevalence of 0.5% (95% CI 0.3–1.5) in urban areas and 0.2% (95% CI 0.05–0.3) in rural areas although the difference was not statistically significant (*P* = 0.063). Most of the GS cases detected in the study were high school or university students. History of parental consanguinity was reported in 67% of enrolled GS subjects. The mean age of the subjects with GS was 29.24 years (range 16–43 years). Of the study subjects, 12 have been previously diagnosed with GS while the rest were diagnosed when enrolled in the study.

**Table 2:** Several significant differences in the clinical presentation existed between enrolled subjects with Gilbert syndrome and health controls

Parameters	*Group A* Patients with Gilbert syndrome (*n* = 83)	*Group B* Patients with GS and eradicated HCV (*n* = 18)	Controls (*n* = 100)	*P* value
Jaundice episodes: number (percent)	83 (100)	18 (100)	0	Group A vs B: 1.000
Frequency of jaundice episodes per year. N (%)				
▪ 1–5	57 (68.674)	10 (55.56)	0	Group A vs B: 1.0000
• 6–10	20 (24.096)	5 (27.76)	0	Group A vs B: 1.0000
• > 10	6 (7.229)	3 (16.67)	0	Group A vs B: 0.7111
Mean duration of individual jaundice episode (mean ± SD) days	19.832 ± 14.	21.1832 ± 15.68	0	Group A vs B: 0.7219
Other symptoms/signs during hyperbilribunemia attacks in GS subjects and control subjects; n (%)				
Fatigue: number (%)	36 (43.37)	4 (22.22)	2(2)	Group A vs. B: 0.1163 Group A vs controls: < 0.0001** Group B vs controls: < 0.0001**
Abdominal pain: number (%)	21 (25.3)	8 (44.44)	3 (3)	Group A vs. B: 0.1490 Group A vs controls: < 0.0001** Group B vs controls: < 0.0001**
Dyspepsia: number (%)	19 (22.89)	7 (38.89)	2 (2)	Group A vs. B: 0.2321 Group A vs controls: < 0.0001** Group B vs controls: < 0.0001**
Bloating; number (%)	17 (20.48)	9 (50)	10(10)	Group A vs. B: 0.0159* Group A *ys*. Controls: 0.0595* Group B vs. controls: 0.002*
Loss of appetite: number (%)	10 (12.048)	2 (11.111)	13 (13)	Group A vs. B:0.6125 Group A *ys*. Controls: 1.0000 Group B vs. controls: 1.0000
Comorbid conditions				
• Diabetes mellitus (Type 1)	4 (4.82)	0	0	Group A vs B: 1.0000
• Diabetes mellitus (Type 2)	2 (2.41)	(11.11)	0	Group A vs B: 0.1449
• Hypertension	1 (1.2)	2 (11.11)	0	Group A vs B: 0.0811
• Rheumatoid arthritis	2 (2.41)	0	0	Group A vs B: 1.0000
• Rheumatic heart valve disease	1 (1.2)	0	0	Group A vs B: 1.0000
Food intolerances and allergies: Celiac/lactose intolerance/food allergies	9 (10.84)	1 (5.56)	3 (3)	Group A vs B: 0.6859 Group A vs. controls: 0.0391* Group B vs. controls: 0.4890
Factors associated with attacks of clinical jaundice n (%)				
Prolonged fasting > 12 h/Ramadan fasting	60 (72.289)	14 (77.78)	0	Group A vs. B: 0.7736
Heavy physical exercise	25 (30.120)	4 (22.22)	0	Group A vs. B: 0.5788
Menstrual abnormalities in women	7 (8.43)	3 (16.67)	0	Group A vs. B: 0.3779
Pregnancy (in 8 women)	8/8 (100)	0	10 (10)	
Cesarean delivery	4 (4.82)	0	0	Group A vs. B: 1.0000
Systemic infections	21 (25.301)	9 (50)	0	Group A vs. B: 0.0486*
Surgery	27 (32.530)	2 (11.11)	0	Group A vs. B: 0.0873
Diets reported as potentially related to jaundice episodes; n (%)				
Drugs (N;%)				
-Paracetamol	2 (2.4)	1 (22.22)	0	Group A vs. B: 0.6186
-Antibiotics(amoxicillin/clavulanate; ofloxacin)	7 (8.434)	2 (11.11)		Group A vs. B: 0.0500*
-Direct acting anti-viral agents	0	7 (38.889)	0	Group A vs. B: 0.6600
-*Diclofenac*	3 (3.6)	1 (16.67)	0	Group A vs. B: 0.1493
-Oral hypoglycemic drugs	1 (1.2)	0	0	Group A vs. B: 0.3261
-Anti-hypertensive drugs	0	0	0	Group A vs. B: 0.1782
Diets reported as potentially related to jaundice episodes; n (%)				
High animal proteins rich/ Fat rich diets	4 (4.82)	0	0	

*P* values < 0.05 are significant

* significant *P* value

** Highly significant statisitcal statistical difference

Recurrent jaundice episodes was the only manifestation in 47/83 (56.627%) subjects; while jaundice was associated with other symptoms such as fatigue, abdominal pain, dyspepsia and loss of appetite in 36 (43.37%), 21 (25.23%), 19 (22.89) and 17 (20.48%) of GS subjects respectively. Individual overt jaundice episodes ranged between 32 and 57 days with a mean of 19.832 ± 14.381 and 21.1832 ± 15.68 days (*p* = 0.7219) in patients with GS alone or patients with GS and patients with GS and treated HCV respectively. In 72.289% of enrolled subjects, overt hyperbilirubinemia was observed with prolonged fasting (> 12 h) particularly during the month of Ramadan when Muslims completely abstain from food and drink daily for more than 16 h extending from before sunrise through sunset for 30 days. Clinical jaundice episodes were reported by 13 (12.871%) individuals with GS after performing high intensity sports such as soccer, tennis, fast swimming or weight lifting, however, the emergence of hyperbilirubinemia was coupled in most cased with low calorie diets. Other risk factors for overt jaundice reported by the study subjects included prolonged infections (25.301%), surgery with total anesthesia (32.53%) and extensive dental procedures (45.783%). Postoperative jaundice occurred in 3 patients with GS after appendectomy, splenectomy**,** and hemorrhoids surgery in addition to 3 women undergoing Cesarean delivery. All surgeries were performed under general anesthesia and were uneventful. Three out of the six patients have been already diagnosed with GS while 3 were diagnosed through investigations conducted after the emergence of jaundice. The pre-operative unconjugated bilirubin ranged between 1.8 mg/dl and 3.6 mg/dl and the post-operative unconjugated bilirubin escalated the second day of surgery to range between 2.9 mg/dl and 4.6 mg/dl then started to decline after one week. In contrast, two GS subjects in the study cohort did not develop jaundice exacerbation following surgeries (perianal abscess and hemorrhoids) performed under spinal anesthesia (Table 2).

In Group B, twelve subjects (66.667%) of whom 4 patients were not known cases of GS, reported development of significant jaundice after initiation of direct antiviral therapy direct (DAAs) with or without ribavirin. The jaundice persisted during DAA treatment despite normalization of the liver transaminases, clearance of HCV RNA through therapy and absence of other adverse events. Following sustained virologic response (SVR), 5/12 subjects developed episodes of unconjugated hyperbilirubinemia (Table 2).

#### Gilbert syndrome characteristics in women

Although GS was more frequent in men compared to women (Table 2), the frequency and duration of jaundice episodes were slightly higher compared to men; although the difference in not statistically significant (15.31 ± 9.24 .in men and 18.51 ± 11.38 in women; *P* = 0.083) *(data not shown).* In women, clinically evident unconjugated hyperbilirubinemia associated certain menstruation abnormalities such as dysmenorrhea and metrorrhagia (43.75%), use of oral contraceptive pills (25%), cesarean delivery (37.5%). Interestingly, pregnancy was associated with exacerbation of unconjugated hyperbilirubinemia in all women who became pregnant during the study (*n* = 8). Elevated unconjugated bilirubin levels were detected at 10–16 pregnancy weeks’ and remained high throughout pregnancy and part of the post-partum period. Other than jaundice, the course of pregnancy was uneventful. Cesarean section was recommended due to obstetric causes in 4 (50%) of the study pregnant women with GS. Seven of the newborns had neonatal hyperbilirubinemia.

#### Comorbidities with GS

Twenty of the 101 (19.801%) subjects with Gilbert Syndrome (groups A and B) had comorbid conditions such as diabetes mellitus, rheumatoid arthritis, and hypertension with rates similar to general population (Table 2). However, the frequency of food intolerance disorders such as celiac and lactose intolerance was significantly higher among subjects with GS compared to control subjects (*P* = 0.0391) (Table 2).

#### Biochemical and nutritional data

**Fig. 1:**
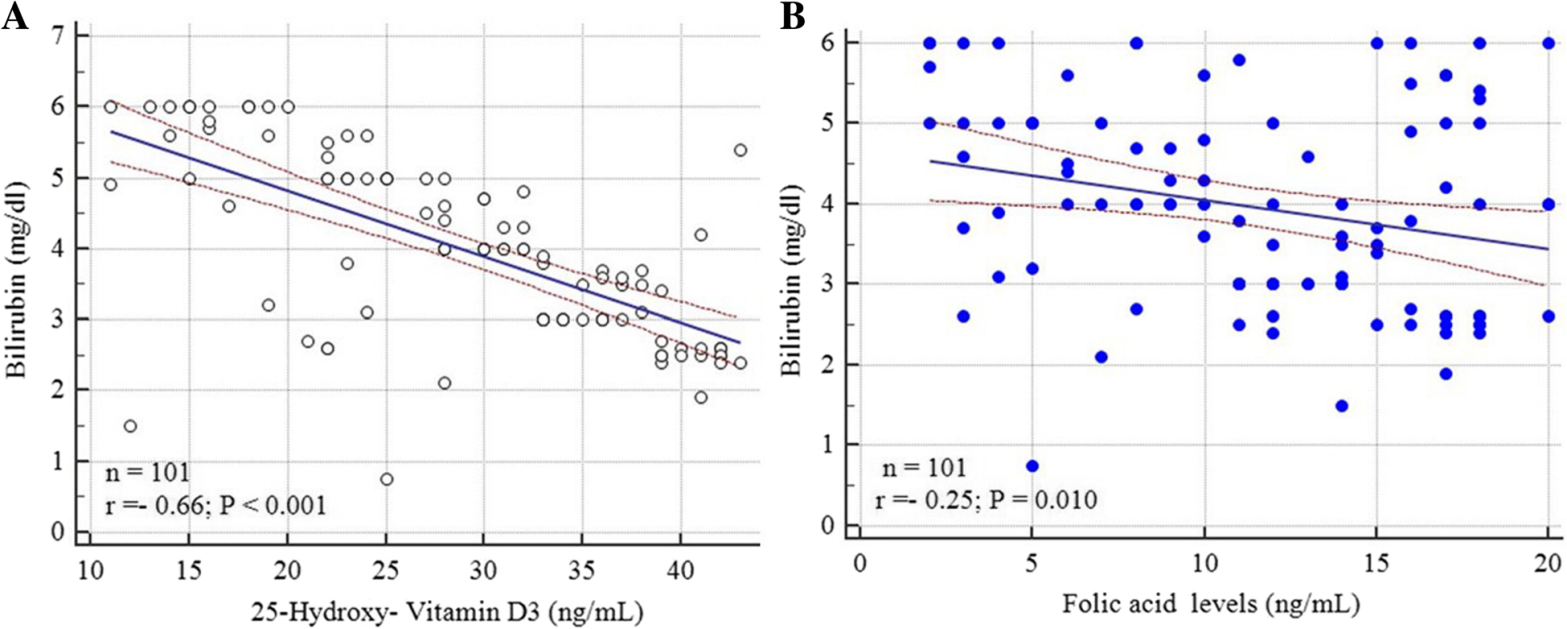
Correlation of Bilirubin levels (mg/dl) with: (**a**) 25-hydroxy-Vitamin D3 (ng/mL) and (**b**) Folic acid levels (ng/mL). Significant correlations was observed between bilirubin levels and each of 25-hydroxy-Vitamin

Individuals with GS without and with treated HCV had significantly lower levels of very low density lipoproteins (VLDL) and intermediate density lipoproteins (IDL). (*P* = 0.044; Table 3). A significant difference was detected in vitamin D levels between GS patients and control subjects (Table 2) with significant inverse correlation between 25-hydroxy-vitamin D3 levels and bilirubin levels (r = − 0.696, *P* = < 0.001; 95% CI: -0.785 to − 0.580) (Fig. 1a). Similarly folic acid levels significantly differed between subjects with GS and controls with an inverse correlation between bilirubin levels and folic acid levels (Table 3 and Fig. 1b). No significant difference was in vitamin B12 levels was detected between GS subjects and control subjects (Table 3).

**Table 3:** Laboratory data of enrolled GS subjects during and in between the jaundice episodes in comparison to control subjects

Parameters	*Group A* Patients with Gilbert syndrome (*n* = 83)	*Group B* Patients with GS and eradicated HCV (*n* = 18)	Controls (*n* = 100)	*P* value
Mean liver function tests in between jaundice episodes				
Total Bilirubin (mg/dL) Mean ± SD (range)	0.42 ± 0.4 (0.3–1.1)	0.61 ± 0.5 (0.5–1.2)	0.38 ± 0.3 (0.1–1.2)	Group A vs. Controls: 0.4410 Group B vs. controls: 0.0087* Group A vs. B: 0.0842
Unconjugated Bilirubin (mg/dL) Mean ± SD (range)	0.21 ± 0.27 (0.1–0.38)	0.41 ± 0.29 (0.2–0.41)	0.17 ± 0.19 (0.1–0.38)	Group A vs. Controls: 0.2425 Group B vs. controls: 0.0096* Group A vs. B: 0.1628
ALT (U/L) Mean ± SD (range)	20.16 ± 4.83 (18–28)	24.06 ± 6.18 (19–37)	19.86 ± 6.31 (17–36)	Group A vs. Controls: 0.7220 Group B vs. controls: 0.0102* Group A vs. B: 0.0038*
AST (U/L) Mean ± SD (range)	23.18 ± 4.83 (17.14–38.5)	24.72 ± 6.82 (21.63–38.24)	22.15 ± 4.06 (17.3–32.63)	Group A vs. Controls: 0.1188 Group B vs. controls: 0.0301* Group A vs. B: 0.4770
Alkaline Phosphatase (U/L) Mean ± SD (range)	80.36 ± 10.85 (75.81–106.17)	91.04 ± 10.74 (80.38–114)	81.16 ± 19.27 (7.45–91.5)	Group A vs. Controls: 0.7369 Group B vs. controls: 0.0001** Group A vs. B: 0.0003*
Total Protein (g/L) Mean ± SD (range)	77.26 ± 5.39 (71.37–83.97)	76.95 ± 4.85 (70–82.96)	77.84 ± 5.83 (72.74–84.67)	Group A vs. Controls: 0.4891 Group B vs. controls: 0.5429 Group A vs. B: 0.8225
Albumin (g/dl) Mean ± SD (range)	4.1 ± 0.53 (3.6–5.4)	3.99 ± 0.61 (3.46–5.2)	4.2 ± 0.75 (3.76–5.5)	Group A vs. Controls: 0.3085 Group B vs. Controls: 0.2643 Group A vs. B: 0.4391
Mean liver function tests during jaundice episodes				
Total Bilirubin (mg/dl) Mean ± SD (range)	3.56 ± 1.82 (1.46–5.7)	4.23 ± 2.01 (2.04–7.97)	0.41 ± 0.31 (0.3–1.4)	Group A vs. B: 0.1677 Group A *ys*. Controls: < 0.0001 Group B vs. controls: < 0.0001
Unconjugated Bilirubin (mg/dl) Mean ± SD (range)	2.07 ± 0.96 (1.7–3.6)	3.03 ± 1.08 (1.9–2.06)	0.37 ± 0.11 (0.1 to 0.6)	Group A vs. B: 0.0001** Group A *ys*. Controls: < 0.0001 Group B vs. controls: < 0.0001
ALT (U/L) Mean ± SD (range)	25.59 ± 5.23 (19–39)	29.51 ± 8.06(23–39)	27.4 ± 4.5 (18–36)	Group A vs. B: 0.0110* Group A *ys*. Controls: 0.0083 Group B vs. controls: 0.1360
AST (U/L) Mean ± SD (range)	27.8 ± 5.4 (22–35)	27.59 ± 6.14 (19–37)	29.4 ± 4.7 (20–37)	Group A vs. B: 0.6308 Group A *ys*. Controls: 0.16 Group B vs. controls: 0.1589
Alkaline Phosphatase (U/L) Mean ± SD (range)	84.2 ± 15.9 (77–123)	86.25 ± 17.37 (75–131)	85.9 ± 14.36 (45–97)	Group A vs. B: 0.6267 Group A *ys*. Controls: 0.4488 Group B vs. controls: 0.9268
Total Proteins (g/L) Mean ± SD (range)	74.81 ± 4.7 (71.65–82.9)	71.21 ± 3.9 (6.259–81.85)	73.63 ± 4.41 (71.25–80.2)	Group A vs. B: 0.0026* Group A *ys*. Controls: 0.2096 Group B vs. controls: 0.0510
Serum Albumin (g/dl) Mean ± SD (range)	4.1 ± 0.61 (3.7–5.5)	3.89 ± 0.47 (3.5–5.3)	4.2 ± 0.59 (4.0–5.45)	Group A vs. B: 0.1729 Group A *ys*. Controls: 0.2625 Group B vs. controls: 0.0371*
Hemoglobin, lipids and vitamins during jaundice episode				
Hemoglobin (gm/dl); mean ± SD	13.31 ± 1.97 (11.83–13.74)	12.19 ± 1.38 (12.16–14.14)	13.61 ± 2.01 (12.77–15.37)	Group A vs. B: 0.5414 Group A *ys*. controls 0.3140 Group B vs. controls: 0.226
Cholesterol (mg/dL) Mean ± SD (range)	169.85 ± 23.74 (132.72–175.92)	171.52 ± 27.84 (145.73–185.-14)	175.02 ± 20.14 (142.61–194.98)	Group A vs. B: 0.7909 Group A *ys*. controls: 0.112 Group B vs. controls: 0.526
Triglycerides (mg/dL) Mean ± SD (range)	139.37 ± 24.72 (127.29–240.84)	140.13 ± 31.52 (136.4–151.81)	142.817 ± 20.38 (136.87–159.74)	Group A vs. B: 0.9111 Group A *ys*. controls: 0.302 Group B vs. controls: 0.639
Low-density lipoprotein cholesterol (LDL-C) (mg/dL) Mean ± SD (range)	68.12 ± 22.81 (48.98–78.81)	70.32 ± 21.66 (54.26–76.82)	75.85 ± 22.87 (58.62–103.84)	Group A vs. B: 0.709 Group A *ys*. controls: 0.023* Group B vs. controls: 0.999
High-density lipoprotein cholesterol (HDL-C) (mg/dL) Mean ± SD (range)	54.41 ± 12.72 (45.12–52.83)	57.92 ± 13.42 (40.18–64.08)	56.71 ± 13.85 (52.52–67.17)	Group A vs. controls: 0.300 Group A *ys*. controls: 0.247 Group B vs. controls: 0.733
^a^Vitamin B12 (pg/ml); mean ± SD (95% CI for the mean)	638. 21 ± 142.15 (507.17 to 769.25)	649. 17 ± 163.42 (467.90 to 730.44)	654.71 ± 195.82 (615.86 to 793.57)	Group A vs. B: 0.7733 Group A *ys*. controls: 0.52 Group B vs. controls: 0.91
^b^25-hydroxy-vitamin D3 (ng/mL); mean ± SD; (95% CI for the mean)	21.25 ± 15.89 (18.78 to 35.72)	20.72 ± 11.39 (17.05 to 45.39)	45.82 ± 17.38 (38.37 to 49.27)	Group A vs. B: 0.89 Group A *ys*. controls: < 0.0001 ** Group B vs. controls: < 0.0001**
^c^Folic acid (ng/mL); mean ± SD (95% CI for the mean)	16.53 ± 3.63 (13.95 to 15.1)	15.93 ± 2.51 (14.38 to 16.67)	18.36 ± 3.16 (17.33 to 20.1)	Group A *ys*. B: 0.4073 Group A vs. controls: < 0.0002** Group B vs. controls: < 0.0001**

^a^Vitamin B12 normal range: 200 to 900 picograms (pg) per milliliter. Normal B12 status**:** > 550 pg/ml and deficient in B12: < 550 pg/ml

^b^25-hydroxy-vitamin D normal range: 20 to 60 ng/mL. Levels less than 20 ng/mL suggest vitamin D deficiency

^c^Folic acid normal range in adults: 2–20 ng/mL

*P* value < 0.05 is significant

* *P* value is significant

** *P* value is highly significant

#### Health related quality of life assessment

Between the episodes of jaundice, almost all SF-36v2, SF-6D and CLDQ scores and domains were comparable between individuals with GS and normal Egyptian population except for fatigue. During hyperbilirubinemia episodes, subjects with GS had significant differences in several domains such as SF-6D preference, vitality, role emotional, social functioning, fatigue, worry and general health (Table 4).

**Table 4:** Mean (standard deviation) of Short form-36 Health Survey version 2 and CLDQ in the two groups during and between episodes of unconjugated hyperbilirubinemia

Scores (norm)*	Subjects with Gilbert Syndrome) (101 subjects; *n* = 83 with GS and 18 with GS and treated HCV)		Control subjects (*n* = 100)	*P* value	
	During jaundice episode	In between jaundice episodes		During jaundice episode	In between jaundice episodes
SF-6D preference	0.72 ± 0.12	0.77 ± 0.12	0. ± 0.11	< 0.0001**	0.278
*SF-36v2*					
PF	82.5 ± 7.35	89.952 ± 2.41	90.10 ± 2.40	0.0001**	0.051
RP	90.19 ± 4.2	89.19 ± 4.2	90. ± 1.4	0.06	0.81
BP	81.3 ± 3.5	83.03 ± 2.8	82.63 ± 6.36	0.07	0.56
GH	49.27 ± 14.5	52.64 ± 10.3	53.24 ± 9.11	0.02 *	0.66
VT	55.34 ± 4.61	59.81 ± 4.18	60.35 ± 3.617	0.0001**	0.33
SF	87.16 ± 9.73	92.74 ± 15.2	92.47 ± 14.9	0.0031**	0.9
RE	80.61 ± 7.24	88.39 ± 5.21	88.53 ± 8.56	< 0.0001**	0.88
MH	71.9 ± 3.6	72.6 ± 3.04	72.10 ± 4.3	0.38	0.71
PCS	45.00 ± 12.9	47.927 ± 9.21	48.81 ± 8.7	0.02	0.48
MCS	49.81 ± 3.9	49.71 ± 3.82	50.87 ± 4.31	0.07	0.07
*CLDQ*					
AS	5.48 ± 1.83	6.2 ± 1.64	6.23 ± 1.55	0.012*	0.58
FA	4.80 ± 1.25	5.37 ± 1.36	5.72 ± 1.03	< 0.0001**	0.04*
SS	5.02 ± 0.82	5.712 ± 1.224	5.82 ± 1.0	< 0.0001 **	0.47
AC	5.87 ± 1.84	6.25 ± 1.33	6.55 ± 1.41	0.004 **	0.12
EF	5.231 ± 1.74	5.73 ± 1.41	5.84 ± 1.43	0.007 *	0.59
WO	5.41 ± 1.26	5.83 ± 1.54	5.90 ± 1.632	0.02*	0.72
Overall	5.32 ± 1.36	5.82 ± 0.89	5.92 ± 1.63	0.006**	0.64

*GS* Gilbert syndrome, *HCV* hepatitis C virus infection

*Significant difference between the two study groups (*P* < 0.05). ** Highly significant difference between the two study groups during the jaundice attack or between jaundice attacks (*P* < 0.01, 0.001)

*AC* activity, *AS* abdominal symptoms, *BP* bodily pain, *EF* emotional function, *FA* fatigue, *GH* general health, *MCS* Mental Component Summary Score, *MH* mental health, *PCS* Physical Component Summary Score, *PF* physical functioning, *RE* Role Emotional, *RP* role physical, *SF* social functioning, *SS* systemic symptoms, *VT* vitality, *WO* worry

During jaundice episodes, significant differences were observed between subjects with GS ans controls in: SF-6D, the *SF-36v2* domains: PF, GH, VT, SF. RE and all CLDQ domains.

In between the hyperbilirubinemia attacks no significant differences in SF-6D, SF-36v2 domains or CLDQ domains except for a slight difference in FA

#### UGT1A1 alleles and genotype frequencies

The UGT1A1 gene variants were analyzed and identified according to the number of (TA) repeats in the promoter TATA box. In the current study, the wild type was UGT1A1 6 T allele which was restricted to control subjects in addition to the variants 5 TA, 7TA and 8TA (Table 5). Analysis of the (TA) polymorphism in the UGT1A1 gene promotor showed that homozygosity for UGT1A1^*^28 was the most frequent finding in subjects with Gilbert’s syndrome (Groups A: 97.546% and B: 95.238%; *P* = 0.6046) (Table 5). In control subjects, homozygous 6/6 UGT1A1 genotype (wild type) was the most prevalent genotype (75%) followed by the heterozygous genotypes 6/7 (12%), 5/7 (11%), 6/8 (1%) and 7/8 (1%)**.** GS subjects with heterozygous UGT1A1 mutations tend to have higher bilirubin. Levels compared with homozygotes (Table 4).

**Table 5:** Genetic variations detected in in the study cohorts

Gene polymorphism	Allele status	Total	Individuals with recurrent episodes of overt jaundice (GS: *n* = 83)	Individuals with GS and eradicated HCV (*n* = 18)	Control subjects (*n* = 100)
UGT1A1*6					
SNP 211G > A; (n,%)	−/−	189	80 (96.386)	12 (66.667)	97 (97)
	*6/−	10	2 (2.409)	5 (27.778)	3 (3)
	*6/*6	1	0	1 (5.555)	0
UGT1A1*27					
SNP 686C > A; (n,%)	−/−	199	81 (97.59)	18 (100)	100 (100)
	*27/−	2	2(2.409)	0	0
	*27/*27	0	0	0	0
UGT1A1*28					
TA repeat A(TA)_7_TAA; (n,%)	−/−	100	0	0	100
	*28/−	3	2 (2.409)	1(5.555)	0
	*28/*28	98	81(97.59)	17 (94.444)	0
UGT1A1*60					
SNP -3263 T > A	−/−	189	71	13	95
	*60/−	13	5	3	5
	*60/*60	9	7	2	0

*GS* Gilbert syndrome, *HCV* hepatitis C virus infection, *UGT1A1* Uridine diphosphoglucuronate-glucuronosyltransferase *A1 SNP*s ingle nucleotide polymorphism

* Allele designation

#### Risk factors associated with overt clinical jaundice attacks

The multivariate logistic regression analysis for development of clinical jaundice episode showed that fasting more than 12 continuous hours particularly religious fasting in the month of Ramadan, low calorie intake (< 1200 cal), general anesthesia, high intensity sports, menstrual abnormalities, pregnancy, and Caesarian delivery have been identified as risk factors for exacerbation of unconjugated hyperbilirubinemia and clinical jaundice in subjects with GS (Table 6).

**Table 6:** Logistic regression analysis revealed that fasting more than 12 hours, adoption of low calories weight losing plans, pregnancy, intensive sports, surgery including Caesarian delivery and severe systemic infections were risk factors for clinical jaundice and hyperbilirubinemia in subjects with Gilbert syndrome

Variables	Odds ratio	CI (95%)	*P* value
Fasting > 12 h	1.973	1.24–2.76	0.002**
Low calories weight losing plans	2.275	1.10–4.68	0.026*
Fat rich diets	1.417	0.93–2.14	0.099
Intensive physical exercise and sports	2.737	1.12–6.69	0.027*
Menorrhagia or metrorrhagia or dysmenorrhea	2.898	1.05–7.95	0.0318*
Pregnancy	4.746	1.65–13.64	0.004**
Vaginal delivery	2.335	0.79–6.84	0.122
Caesarian delivery	2.110	1.07–4.16	0.031**
Surgery under general anesthesia	4.139	1.96–8.71	< 0.001**
Systemic infections	2.174	1.387–2.861	0.0413*

P values < 0.05 are significant

*Significant statistical difference

** Highly significant statistical difference

## Discussion

GS is a clinically ignored, underestimated and insufficiently investigated clinical entity particularly in Egypt where G S has not been previously investigated. Therefore, the current study assessed the frequency, clinical course, genetic parameters of GS through a cross-sectional and longitudinal study design.. The cross-sectional study and analysis of questionnaires showed that the prevalence of GS among the Egyptian population is 8.016% with slight predominance among males which is comparable to the prevalence rates in previous Western studies [7, 28–33]. However, our study showed relatively higher rates in rural settings, a finding that maybe related to higher rates of consanguinity. In the majority of enrolled subjects, clinical jaundice was accidently observed during puberty or early adult life in agreement with previous studies [1, 7, 28] given that some subjects with GS are born with varying amounts of UGT1A1 enzyme activity that may support bilirubin conjugation during early childhood but is not sufficient for complete conjugation during adult life and increased activities [11]. At the onset of clinical jaundice, preliminary diagnosis in the majority of the study cases was viral hepatitis, cholelithiasis or hemoglobinopathy given the prevalence of such diseases in Egypt. Despite exclusion of such conditions, GS was diagnosed in only 25% of the enrolled subjects suggesting that there is poor awareness about GS not only among the public but also among general physicians.

In the current study, 50% of enrolled subjects experienced 1–5 episodes of hyperbilirubinemia per year during which clinical jaundice was the only presentation. However, approximately 50% of the study cohort reported coexistence of jaundice with other symptoms such as abdominal pain**,** dyspepsia and bloating which were also demonstrated during the follow-up visits and were shown by HRQOL measures to have emotional, physical and lifestyle implications on GS subjects.. The cause of such gastrointestinal manifestations is not clear, however, it may be due to disturbed gastric emptying as shown in one study [34] or impaired bilirubin kinetics in GS [35] or insufficiency of the UGT1A1 enzyme which is required for the glucuronidation and elimination of some food metabolites such as N-hydroxy-PhI [36, 37].

In the current study, Ramadan fasting (abstaining from food and drink for 14–16 h daily for 30 days) and following a strict low calorie weight losing plans were strong risk factors for overt clinical jaundice attacks in GS subjects. Follow-up of our GS cohort showed that the levels of bilirubin directly correlated with the interval of fasting or calorie deprivation. A previous study showed exacerbation of unconjugated hyperbilirubinemia by religious fasting [38]. This finding has clinical implications for providing dietary consultation and nutritional advice in subjects with GS planning to follow weight loss programs.

In accordance with previous studies, calories restriction and intensive physical exercise were risk factors for attacks of unconjugated hyperbilirubinemia and clinical jaundice. Logistic regression showed that surgery. Cesarean delivery, pregnancy, menstrual abnormalities, prolonged systemic infections and low calories weight loss plans were significant risk factors for emergence of clinical jaundice episodes. In the current study, postoperative jaundice occurred in 6 patients with GS after surgical interventions conducted under general anesthesia but not under spinal or local anesthesia suggesting the role of anesthesia in provoking hyperbilirubinemia. Peri-operative fasting and analgesics [39, 40] used after surgery may also play a role in exacerbation of bilirubinemia in GS subjects undergoing surgery. The slightly higher prevalence of food intolerance particularly celiac disease in subjects with GS is interesting and needs further studies to validate. A positive history of parental consanguinity was found in the patients suffering of both conditions. Taken together, GS should be considered in per-operative evaluations in patients considered for elective surgeries to tailor the type of anesthesia, post-operative analgesics and minimize the duration of fasting in GS subjects exposed to surgery to avoid exacerbation of postoperative hyperbilirubinemia [41, 42].

The current study explored the course of GS in women compared to men. Although less women were diagnosed with GS in the current study, exacerbations in unconjugated bilirubin were more frequent and slightly more prolonged in women. The study showed that women were exposed to additional risk factors for hyperbilirubinemia particularly menstrual abnormalities, pregnancy and deliveries. The study demonstrated that pregnancy represented an important risk factor for exacerbation of indirect hyperbilirubinemia starting from the first trimester and persistingthrough-out pregnancy and part of the post-partum period. This is an interesting finding which has important clinical implications. GS should be considered in the differential diagnosis of jaundice of pregnancy and discriminated from other liver diseases related to pregnancy such as intrahepatic cholestasis of pregnancy, hyperemesis gravidarum preeclampsia, HELLP syndrome, and acute fatty liver of pregnancy.

Given the high prevalence of hepatitis C infection and the frequency of GS in Egypt as, the coexistence of HCV and GS is expected and designing antiviral therapy for this population is critical. The current study demonstrated marked elevations in bilirubin (mostly unconjugated) in 12 patients with concomitant HCV and GS (some of whom were not previously known to have GS) after initiation of DAAs with or without ribavirin. The history of recurrent jaundice attacks, the predominant unconjugated hyperbilirubinemia, and normalization of the liver transaminases as well as undetectable HCV RNA through therapy and absence of serious adverse events supported the diagnosis of GS and DAA therapy was continued until achievement of sustained virologic response. Some reports [43, 44] raised the issue of management of patients with HCV and GS with DAAs with concerns about the safety of specific DAAs regimen. Further studies are necessary to optimize the management of HCV in patients with coexisting HCV. Gilbert’s syndrome may not be a contraindication to HCV treatment with DAAs but GS needs to be considered in patients’ screening to avoid confusion during monitoring patients during and after therapy.

in subjects with current study showed significant difference in 25-Hydroxyvitamin D3 levels between subjects with GS and control subjects, a finding that has not been previously reported. Although the sample size in the current study may not be sufficient to make conclusions, the differences in vitamin D3 levels may be explained by defective glucuronidation in GS subjects. 25OHD_3_, the major circulating form of vitamin D_3_ in humans, blood concentrations represent a balance between it’s formation and clearance by several processes. Thus, differences in the efficiency of any of these processes, including glucuronidation, may affect circulating plasma concentrations of 25OHD_3_. Some studies identified different glucuronide conjugates of 25OHD_3_ and showed some variants of UGT1A that acted as principal catalysts of 25OHD_3_ glucuronidation in human liver [45]. It is not clear if UGT1A defects may result in vitamin D3 alterations in subjects with GS and if Vitamin D screening is recommended in these subjects.

The current study is particularly concerned with the clinical aspects, risk factors for exacerbation of unconjugated bilirubin and impact of GS on the quality of life rather than extensive genetic analysis. The most common genotype of GS detected among the current study subjects was the homozygous polymorphism A(TA)7TAA which is comparable to genotype reported in Caucasians rather than Asian populations [46, 47]. As previously reported, subjects with hetergenous polymorphisms showed higher levels of bilirubin compared to those with homozygous polymorphism [48, 49]. The significance and implications of such association need to be further investigated.

Gilbert syndrome has long been described is an asymptomatic disorder that does not have clinical implications and no monitoring is recommended. However, the current study showed that the quality of life in GS subjects is affected particularly during the jaundice episodes. Subjects with GS had significant differences in SF-6D preference, vitality, role emotional, social functioning, worry and general health. Some subjects opt from activities during the jaundice attacks either due to symptoms such as jaundice and gastrointestinal discomfort or embarrassment due to lack of awareness of general public about GS.

The current study has several strengths. This is the first study, to our knowledge, that investigates the frequency, clinical course of GS in Egyptians and the risk factors for clinical unconjugated hyperbilirubinemia episodes utilizing a longitudinal and cross sectional. Study design. The study identified several symptoms that occur with jaundice and impair several domains of HRQOL during the unconjugated hyperbilirubinemia episodes. The study described the course of GS during pregnancy and further longitudinal studies are required to investigate the impact of GS on the neonates. We also recognize limitations of our study. First, the sample size may not be sufficient to drive final conclusions and larger cohorts are required to validate some findings such as the decreased vitamin D and folic acid levels in subject with GS and the potential association between GS and food intolerances. The impact of different direct antiviral regimen on hyperbilirubinemia in patients with HCV and GS is an important aspect that needs to be further studied to optimize treatment of chronic HCV in subjects with GS.

## Conclusion

Gilbert syndrome is a frequent but underestimated hereditary disorder in Egypt. GS is an entirely benign condition as thought. GS is associated with clinical manifestations during the hyperbilirubinemia attacks and GS has an impact on the quality of life. Screening for GS is necessary along with raising the awareness of the manifestations of GS. Subjects with GS need to be well informed of the risk factors for exacerbation of hyperbilirubinemia and avoid such factors. Subjects with GS need special care in specific conditions such as surgery, anesthesia, weight loss plans design, prescription of medicines particularly direct acting antivirals.

## Abbreviations

-36v2: Short form-36 v2
AC: Activity
AS: Abdominal symptoms
BP: Bodily pain
CLDQ: Chronic liver disease questionnaire
EF: Emotional function
FA: Fatigue
GH: General health
GS: Gilbert syndrome
HBV: Hepatitis B virus
HCV: Hepatitis C
HRQOL: Health related quality of life
MCS: Mental Component Summary Score
MH: Mental health
NAFLD: Nonalcoholic Fatty Liver Disease
PCS: Physical Component Summary Score
PF: Physical functioning
RE: Role Emotional
RP: Role physical
SF: Social functioning
SS: Systemic symptoms
*UGT1A1*: Uridine disphosphate-glucuronosyltransferase-1A1
VT: Vitality
WO: Worry
